# The solution structure of the heavy chain–only C5-Fc nanobody reveals exposed variable regions that are optimal for COVID-19 antigen interactions

**DOI:** 10.1016/j.jbc.2023.105337

**Published:** 2023-10-12

**Authors:** Xin Gao, Joseph W. Thrush, Jayesh Gor, James H. Naismith, Raymond J. Owens, Stephen J. Perkins

**Affiliations:** 1 Department of Structural and Molecular Biology, Division of Biosciences, University College London, London, United Kingdom; 2 Department of Structural Biology, The Rosalind Franklin Institute, Harwell Science Campus, Didcot, United Kingdom

**Keywords:** analytical ultracentrifugation, antibody, nanobody, atomistic modelling, molecular dynamics, small angle neutron scattering, small angle X-ray scattering

## Abstract

Heavy chain–only antibodies can offer advantages of higher binding affinities, reduced sizes, and higher stabilities than conventional antibodies. To address the challenge of SARS-CoV-2 coronavirus, a llama-derived single-domain nanobody C5 was developed previously that has high COVID-19 virus neutralization potency. The fusion protein C5-Fc comprises two C5 domains attached to a glycosylated Fc region of a human IgG1 antibody and shows therapeutic efficacy *in vivo*. Here, we have characterized the solution arrangement of the molecule. Two 1443 Da N-linked glycans seen in the mass spectra of C5-Fc were removed and the glycosylated and deglycosylated structures were evaluated. Reduction of C5-Fc with 2-mercaptoethylamine indicated three interchain Cys–Cys disulfide bridges within the hinge. The X-ray and neutron Guinier *R*_*G*_ values, which provide information about structural elongation, were similar at 4.1 to 4.2 nm for glycosylated and deglycosylated C5-Fc. To explain these *R*_*G*_ values, atomistic scattering modeling based on Monte Carlo simulations resulted in 72,737 and 56,749 physically realistic trial X-ray and neutron structures, respectively. From these, the top 100 best-fit X-ray and neutron models were identified as representative asymmetric solution structures, similar to that of human IgG1, with good R-factors below 2.00%. Both C5 domains were solvent exposed, consistent with the functional effectiveness of C5-Fc. Greater disorder occurred in the Fc region after deglycosylation. Our results clarify the importance of variable and exposed C5 conformations in the therapeutic function of C5-Fc, while the glycans in the Fc region are key for conformational stability in C5-Fc.

Nanobodies were initially isolated from the serum of the Camelidae family (llamas, alpacas, guanacos, and vicunas) 3 decades ago (1). In these, a subset of IgG-type immunoglobulins was formed from two identical heavy chain–only antibodies) bearing a single variable region (VHH), with no associated light chains, and lacking the C_H_1 domain (Fig. 1*A*) (1). The term “nanobody” was coined by the Belgian company Ablynx because of the nanometric dimensions of an isolated variable VHH domain of size ∼15 kDa (2). Compared to classic 12-domain IgG molecules (∼150 kDa), they are smaller in size (∼90 kDa) but are capable of retaining the binding affinity and specificity of an original full-sized 12-domain IgG antibody. They are valued for their structural stability and ease of engineering into reagents suited for *in vitro* and *in vivo* applications (3).

**Figure 1:**
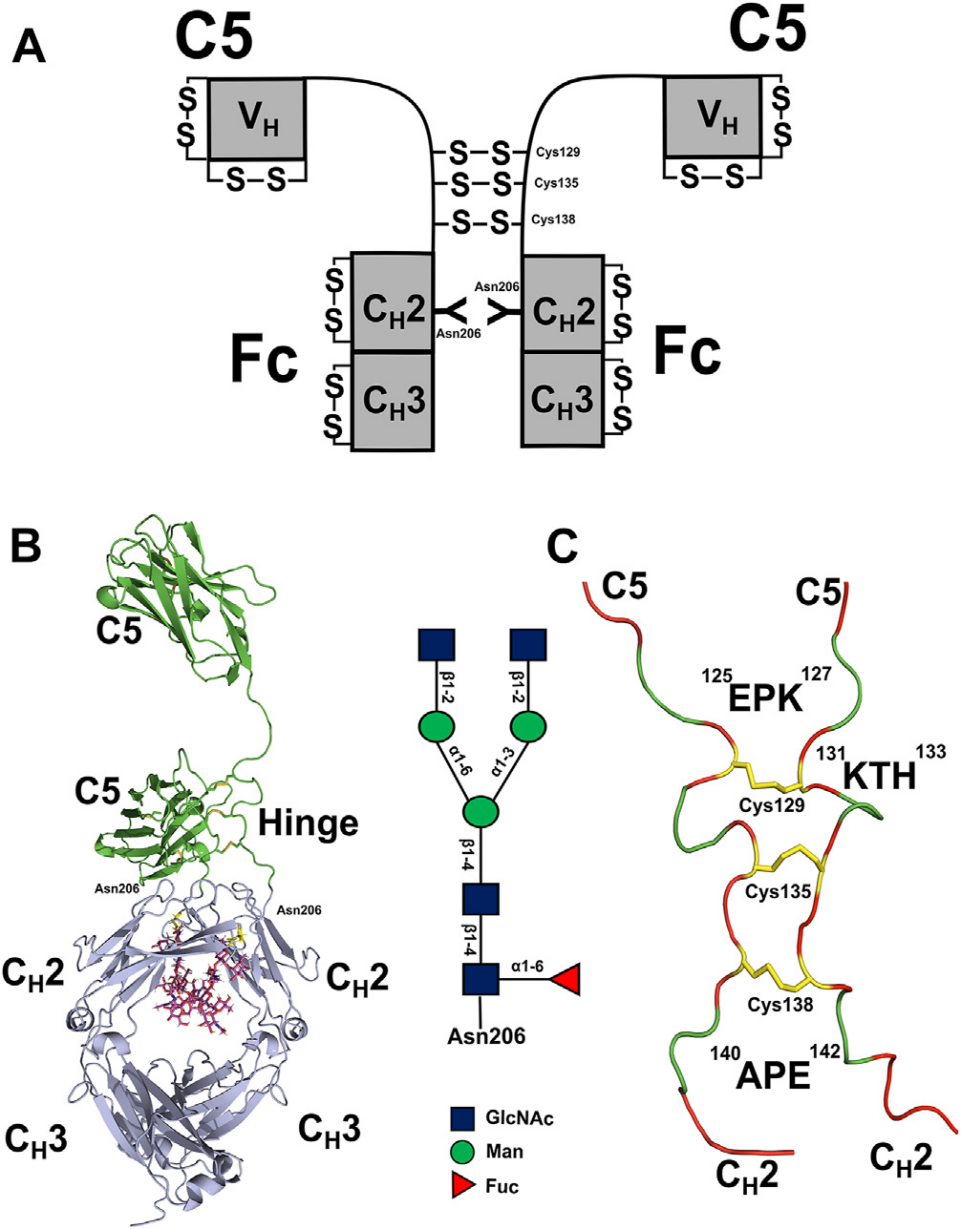
**The domain structure of C5-Fc with its glycans and hinge.** Throughout this study, the monomer form of C5-Fc is defined to have two heavy chains, as depicted. *A,* the C5-Fc structure is composed of two copies of a single-domain V_H_ nanobody C5 that replaces the V_H_ and C_H_1 domains of a conventional antibody heavy chain and the Fc region of human IgG1 with C_H_2 and C_H_3 domains. Cys–Cys disulfide bridges within the C5 and Fc regions are denoted by S–S. Three interchain Cys–Cys disulfide bridges are formed at the central hinges. *B,* C5-Fc is shown as a *ribbon model*, with the two C5 domains in *green* and the Fc region in *slate blue*. The two N-linked glycans are shown in *pink sticks*. The hinge was constructed using the sequence ^122^ASTEPKSCDKTHTCPPCPAPELLGGP^147^. Each N-linked glycan is represented as a *schematic cartoon* (GlcNAc, N-acetyl glucosamine; Man, mannose; Fuc, fucose). *C,* the *close-up view* of the hinge (*red*) shows the six hinge tripeptides ^125^EPK^127^, ^131^KTH^133^, and ^140^APE^142^ (*green*) that were varied in the Monte Carlo searches. The hinges were connected with three disulfide bridges (*yellow*) at Cys^129^, Cys^135^, and Cys^138^.

In the course of developing pharmacological responses to the SARS-CoV-2 coronavirus and the COVID-19 virus pandemic, an extremely potent nanobody with picomolar affinity termed C5 demonstrated high COVID-19 virus strain neutralization activity (4). The isolated C5 nanobody showed high affinity for the receptor-binding domain (RBD) of the SARS-CoV-2 coronavirus spike glycoprotein *in vitro* and competed effectively for the interaction between the viral RBD and host angiotensin-converting enzyme-2 (ACE-2) (4). An immunoglobulin Fc fragment is able to extend the half-life of such a protein *in vivo* after fusion to therapeutic nanobodies (5). Further coupling to an active IgG1 Fc enables engagement with effector functions through interactions with the three classes of human Fcγ receptors (FcγRs) and with the globular heads of human complement C1q. Here, the recombinant fusion protein C5-Fc, comprising two llama-derived single-domain antibodies C5 and a glycosylated Fc region of a human IgG1 antibody was produced in mammalian cells (Fig. 1*A*). In a hamster model of COVID-19 virus, C5-Fc showed therapeutic efficacy *in vivo,* following administration of a single intraperitoneal dose of this heavy chain–only antibody (4). Although the crystal structure of RBD complexed with the isolated monomeric C5 nanobody has been determined (4), the molecular structure of the actual C5-Fc molecule has not been studied. This leaves important questions about the molecular orientation of the two C5 regions in unbound C5-Fc, how C5-Fc interacts with the RBD spike protein in their complex, the structural consequences of the N-linked glycan in the Fc region of C5-Fc, and the structure of the disulfide-linked hinge (Fig. 1, *B* and *C*). An improved understanding of these issues will clarify how C5-Fc is able to block binding of the RBD to ACE-2 and inform future anti-COVID strategies.

To address these questions, we applied small-angle X-ray scattering (SAXS), small-angle neutron scattering (SANS), and analytical ultracentrifugation (AUC) to full-length C5-Fc (Figs. 1 and 2) (6) in physiological concentrations and buffered solutions. Our SAXS and SANS measurements of C5-Fc in light water and heavy water buffers, respectively, provided complementary data sets measured in high-positive and high-negative solute-solvent contrasts (7). The development of atomistic modeling of the SAXS and SANS datasets using molecular dynamics and Monte Carlo (MC) methods, recently discussed in (8), is a powerful new tool for structural studies. To complete our understanding of the C5-Fc structure, we also investigated by mass spectrometry its enzymatic cleavage with PNGase to remove its N-glycans, and the labeling of C5-Fc with maleimide-polyethylene glycol to verify the presence of the hinge disulfide bridges. Here, our combined SAXS-SANS-AUC datasets in conjunction with atomistic modeling demonstrated the variability of the exposed C5-Fc conformations with and without glycans. The results show how C5-Fc behaves in solution as a model for heavy chain–only antibodies, both synthetic and natural, and provides insight into its therapeutic efficacy in binding to the RBD.

**Figure 2:**
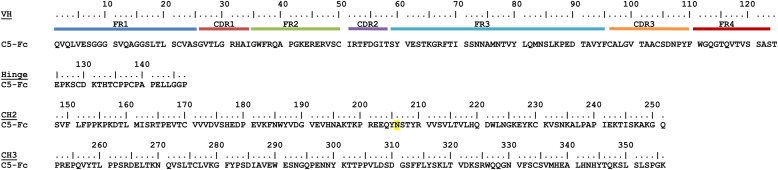
**The C5-Fc sequence.** The C5 sequence corresponds to a V_H_ domain (modeled using PDB ID: 7OAO) and is connected to a human Fc region (modeled using PDB ID: 1HZH) *via* its IgG1 hinge sequence.

## Results

### Purification and characterization of glycosylated and deglycosylated C5-Fc

**Figure 3:**
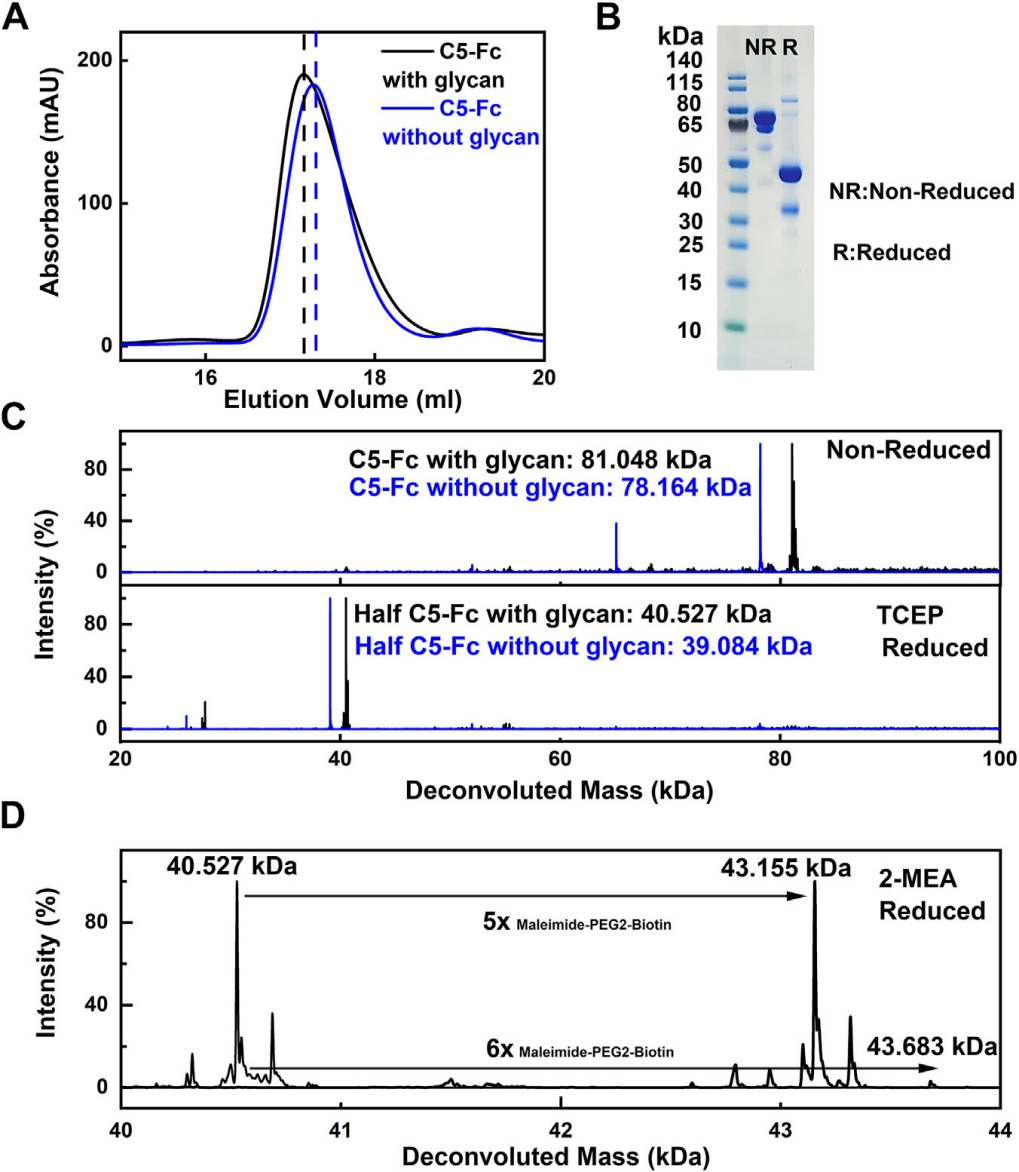
**Purification and characterization of C5-Fc.***A,* elution peaks from a Superose 6 Increase 10/300 gel filtration column for C5-Fc (*black*) and deglycosylated C5-Fc (*blue*) in 10 mM L-histidine, 138 mM NaCl and 2.6 mM KCl, pH 6. The peak positions are indicated by *dashed vertical lines*. *B,* SDS-PAGE of purified C5-Fc. *Lane 1*, molecular mass markers are denoted in kDa to the *left*. *Lanes 2 and 3* correspond to nonreduced and reduced C5-Fc, respectively. *C,* mass spectra of nonreduced and tris(2-carboxyethyl)phosphine-reduced C5-Fc (*black*) and deglycosylated C5-Fc (*blue*). Each of the two glycan chains has a mass of 1443 Da. *D,* mass spectra of monoethanolamine (2-MEA)-reduced C5-Fc before (*left*) and after (*right*) the labeling of its free sulfhydryl groups with a maleimide-polyethylene glycol-biotin label of mass 525 Da. 2-MEA reduced the interchain disulfide bonds inside the antibody hinge.

The recombinant llama-human fusion protein C5-Fc (Fig. 2) was purified in high yields by transient expression in mammalian cells (Experimental procedures). As required, the purified protein was deglycosylated by treatment with peptide:N-glycosidase F (PNGase F) according to the manufacturer’s protocol (Experimental procedures). Initially, gel filtration and SDS-PAGE were used to examine the integrity of C5-Fc, and the protein with and without glycans was eluted as single symmetrical peaks at 17.16 ml and 17.27 ml, respectively (Fig. 3*A*). Native C5-Fc from gel filtration was loaded onto a 4 to 12% Bis Tris NuPAGE SDS-PAGE gel with and without tris carboxy ethyl phosphine (TCEP) as the reducing agent (Fig. 3*B*). The SDS-PAGE gel showed the expected results, indicating the level of purity. Nonreduced C5-Fc showed a major band at 80 kDa, which agreed with the expected mass of 80 kDa calculated from the C5-Fc sequence and a minor band at 65 kDa. Reduced C5-Fc migrated at 40 to 50 kDa to correspond to the expected half molecule of C5-Fc at 41 kDa, following disulfide bond reduction. Another minor band at 30 to 40 kDa was observed, which may be an Fc-only fragment which also would account for the species at 65 kDa under nonreducing conditions.

LC/MS identified the N-linked glycoforms of C5-Fc (Fig. 3*C*) and the number of connected disulfide bridges in the hinge (Fig. 3*D*). Native C5-Fc showed a single peak with a molecular mass of 81.048 kDa, in close agreement with the expected value of 81.206 kDa, and verifying its high purity. Following PNGase F cleavage for 10 h, which achieves full glycan cleavage in human IgG1 (8), a single peak was again seen with a mass of 78.164 kDa, also in close agreement with the expected value of 78.318 kDa from its sequence. The mass of the two glycan chains was calculated to be 2884 Da each from the difference in values. In corroboration of these values, the masses of TCEP-reduced C5-Fc with and without glycans were 40.527 kDa and 39.084 kDa, giving a difference of 1.443 kDa. These masses agree well with a glycan composition of Man_3_GlcNAc_4_Fuc (mass 1444 Da), comprising a core structure with no NeuNAc.GlcNAc antenna (Fig. 1*B*).

Further, mass spectrometry was used to examine the disulfide bridge structure of the C5-Fc hinge region, which has three potential S–S bridges (Fig. 1*A*). The protein was reduced with 2-monoethanolamine and then incubated with polyethylene glycol-biotin, which would add a mass of 0.525 kDa to any free sulphydryl groups present. The mass of native C5-Fc was 40.527 kDa, and this increased by 2.628 kDa and 3.156 kDa to 43.155 kDa and 43.683 kDa, respectively, after labeling (Fig. 1*D*). This mass difference corresponded to the binding of 5 and 6 maleimide-polyethylene glycol-biotin molecules, indicating that all three presumed S–S bridges (Fig. 1*A*) had formed in native C5-Fc, and these were bound to 5 or 6 labels as expected.

### AUC of glycosylated C5-Fc in light and heavy H_2_O

**Figure 4:**
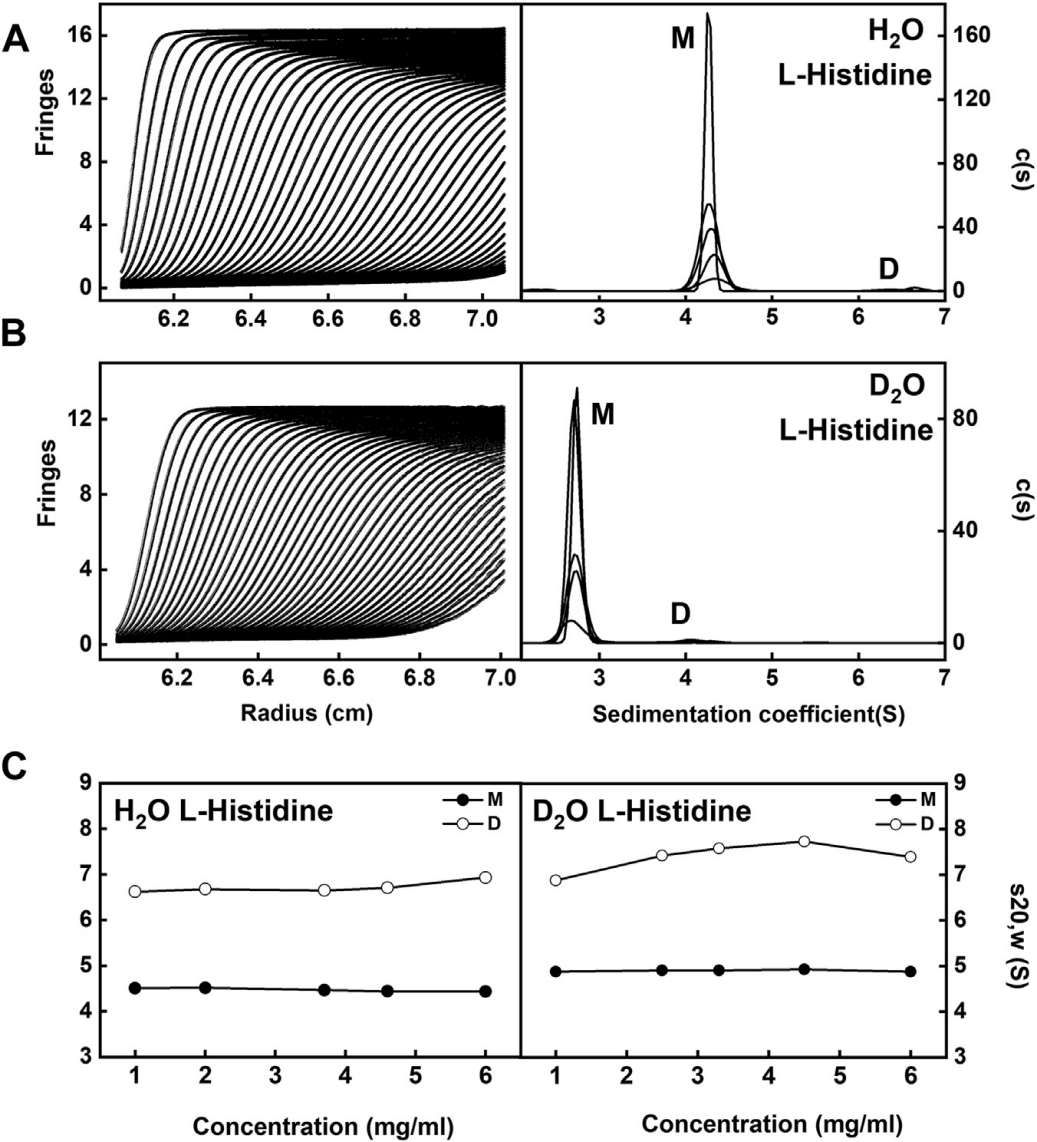
**Sedimentation velocity analyses of C5-Fc in L-histidine buffer.***A,* C5-Fc at 1 mg/ml, 2.5 mg/ml, 3.3 mg/ml, 4.5 mg/ml, and 6 mg/ml, in L-histidine buffer in H_2_O. The SEDFIT boundary fits are shown as *black lines* (*left*). The monomer peak (M) in the size-distribution analyses *c(s)* (*right*) corresponds to sedimentation coefficient *s*_*20,w*_ values of 4.2 to 4.3 S, while a trace dimer peak (D) was visible at 6.4 to 6.8 S. *B,* C5-Fc at 1 mg/ml, 2 mg/ml, 3.7 mg/ml, 4.6 mg/ml, and 6 mg/ml in L-histidine buffer in D_2_O. The SEDFIT boundary fits are shown as *black lines* (*left*). The monomer peak (M) in the size-distribution analyses *c(s)* (*right*) corresponds to *s*_*20,w*_ values of 2.6 to 2.7 S, while a trace dimer peak (D) was visible at 4.0 to 4.3 S. *C,* the concentration dependences of the *s*_*20,w*_ values for the monomer (*filled symbols*) and dimer (*open symbols*) peaks in both buffers are shown. The *s*^*0*^_*20,w*_ value of the C5-Fc monomer is 4.5 ± 0.1 S in L-histidine in H_2_O (*left*). The *s*^*0*^_*20,w*_ value of the C5-Fc monomer is 4.9 ± 0.1 S in L-histidine in D_2_O (*right*).

AUC uses macromolecular sedimentation under a high centrifugal force to determine masses and solution structures (9). This was used to confirm that C5-Fc was well behaved in L-histidine buffer. Five different concentrations of C5-Fc in each of light and heavy water buffers (to correspond to the X-ray and neutron measurements, respectively) were measured by sedimentation velocity runs involving totals of 650 scans. The fits of ∼45 interference boundaries (left panels, Fig. 4, *A* and *B*) in the SEDFIT software (https://sedfitsedphat.github.io/) gave excellent visual agreements between the fitted lines (black line) and experimental scans (circles). The residuals (rms difference) were low at 0.08 to 0.015 fringes. In the resulting size-distribution analyses *c(s)*, native C5-Fc in H_2_O buffer revealed a large peak (M) that corresponded to observed sedimentation coefficient values of 4.2 to 4.3 S for the two heavy-chain monomeric form (Fig. 1*A*), while a trace dimer peak with four chains (D) was visible at 6.4 to 6.8 S (right panel, Fig. 4*A*). The corresponding large peak (M) in the *c(s)* for native C5-Fc in D_2_O buffer corresponds to uncorrected sedimentation coefficient values of 2.6 to 2.7 S, while a trace dimer peak (D) was visible at 4.0 to 4.3 S. The proportion of the C5-Fc monomer was measured at 92 ± 3% in both H_2_O and D_2_O buffers and that for its dimeric form was low at 2.3 ± 0.5%. The change in solvent density and viscosity between light and heavy water causes the observed sedimentation coefficients to differ. The observed sedimentation coefficients were converted to *s*_*20,w*_ values (Fig. 4*C*). The mean *s*_*20,w*_ value of the C5-Fc monomer in H_2_O was 4.47 ± 0.04 S, while that for D_2_O was 4.89 ± 0.02 S (Table 1). No concentration dependence was observed for the monomer *s*_*20,w*_ values in either H_2_O and D_2_O buffers (Fig. 4*C*). Since the corresponding *s*_*20,w*_ value for human IgG1 lies between 6.34 to 6.42 S (8), the smaller *s*_*20,w*_ value for C5-Fc is attributed to its lower mass and more compact size. The mass analyses for the monomer peaks in the *c(s)* plots gave 85 to 91 kDa in both H_2_O and D_2_O buffers, in good accord with the composition-calculated masses of 81 kDa. The 0.42 S increase in the *s*_*20,w*_ value for C5-Fc in D_2_O compared to H_2_O is attributable to changes in the $\bar{v}$ of the hydration shell surrounding C5-Fc, following the replacement of protons with deuterons of twice the mass (10).

**Table 1:** Experimental AUC parameters for C5-Fc

Sample and buffer	Concentration (mg/ml)	s_*20,w*_ (S)^a^	Modeling fit (mg/ml)	100 modeling fits s_*20,w*_ (S)	Best fit s_*20,w*_ (S)
C5-Fc in L-histidine H_2_O buffer	6.00	4.49	5.0	4.22–4.41	4.42
	4.60	4.50	4.0	4.22–4.42	4.35
	3.70	4.52	3.0	4.22–4.43	4.32
	2.00	4.56	2.0	4.25–4.43	4.32
	1.00	4.57	1.0	4.31–4.32	4.31
			0.5	4.25–4.43	4.34
C5-Fc in L-histidine D_2_O buffer	6.00	4.97	6.0	4.13–4.33	4.17
	4.50	5.03	4.5	4.20–4.35	4.24
	3.30	5.00	3.3	4.17–4.33	4.25
	2.50	5.00			
	1.00	4.98			

a Errors on the s_*20,w*_ values were generally 0.01 S.

### SAXS and SANS of glycosylated and deglycosylated C5-Fc

The solution structures of glycosylated and deglycosylated C5-Fc were characterized by SAXS and that of glycosylated C5-Fc by SANS. The two methods differ, in that X-rays in H_2_O buffer detect the hydration shell surrounding C5-Fc, while neutrons in D_2_O buffers reveal a much-reduced view of the hydration shell because of the different solute-solvent contrast in use (7, 11, 12). The two data collections provide tests of self-consistency of each other.

**Figure 5:**
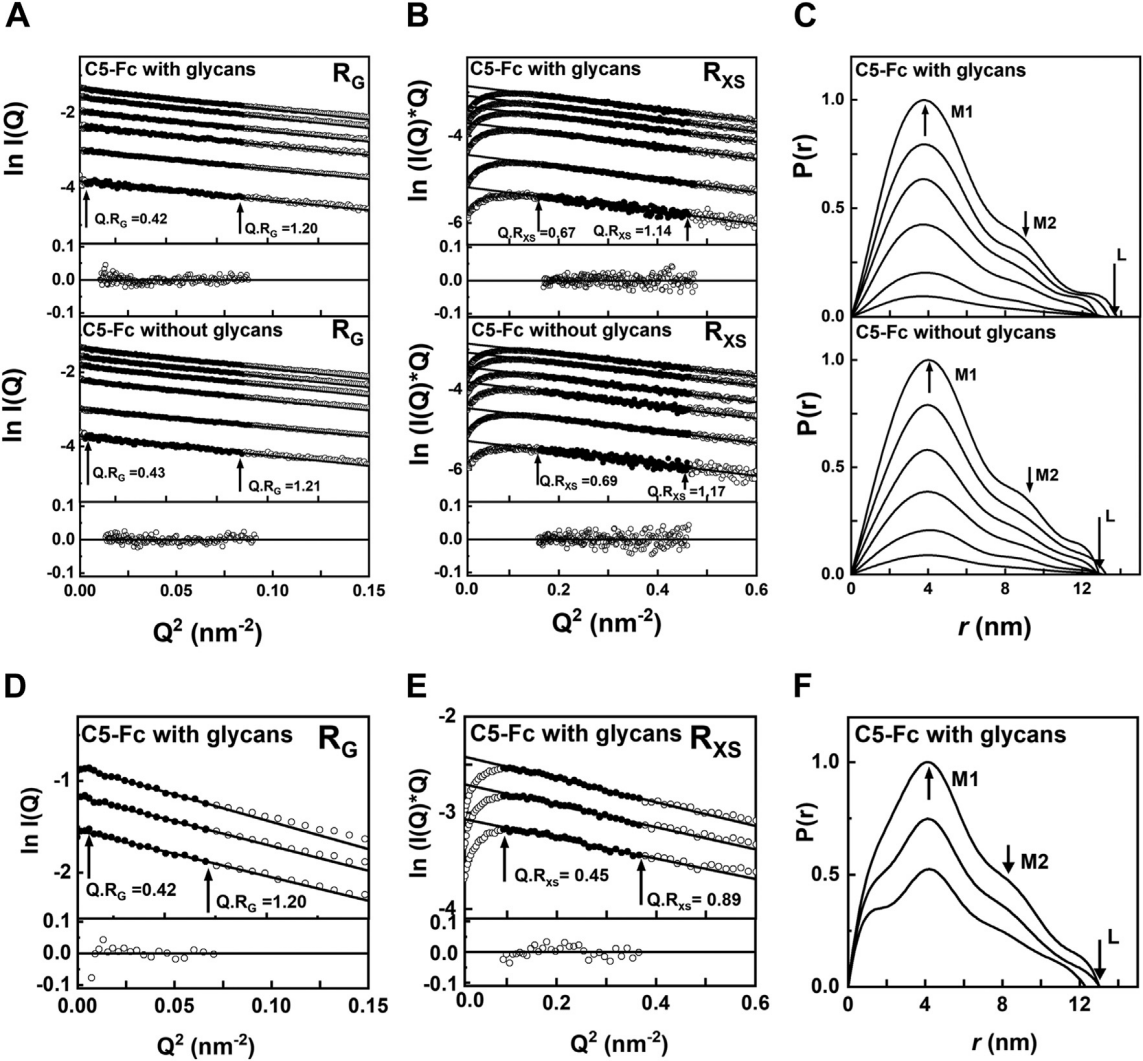
**X-ray and neutron Guinier and distance distribution *P(r)* analyses for C5-Fc.***A,* X-ray Guinier *R*_*G*_ analyses for C5-Fc in L-histidine buffer in light water at 0.5 mg/ml, 1 mg/ml, 2 mg/ml, 3 mg/ml, 4 mg/ml, and 5 mg/ml. The 1 mg/ml sample was run in size exclusion chromatography-SAXS mode while the other concentrations were run in batch mode. The *Q* range was 0.010 to 0.029 nm^−1^. The *filled circles* correspond to the *I(Q)* values used to determine each *R*_*G*_ value. *B,* the corresponding X-ray Guinier *R*_*XS*_ analyses, using a *Q* range of 0.040 to 0.068 nm^−1^. *C,* the corresponding X-ray *P(r)* analyses, where the *arrows* indicate the *M1* and *M2* peaks, and *L* represents the maximum dimension of C5-Fc. *D,* neutron Guinier *R*_*G*_ analyses for C5-Fc in L-histidine buffer in heavy water at 3.3 mg/ml, 4.5 mg/ml, and 6 mg/ml. The *Q* range was 0.0085 to 0.027 nm^−1^. The *filled circles* correspond to the *I(Q)* values used to determine each *R*_*G*_ and *R*_*XS*_ values. *E,* the corresponding neutron Guinier *R*_*XS*_ analyses, using a Q range of 0.03 to 0.06 nm^−1^. *F,* the corresponding neutron *P(r)* analyses, where the *arrows* indicate the *M1* and *M2* peaks and L represents the maximum dimension of C5-Fc.

C5-Fc with and without glycans were studied by SAXS at 20 °C in L-histidine buffer in H_2_O (Experimental procedures) at concentrations from 0.5 to 5.0 mg/ml. The *R*_*G*_ values monitor the overall structure of C5-Fc, while *R*_*XS*_ values monitor the cross-sectional structure of C5-Fc. The Guinier *R*_*G*_ and *R*_*XS*_ analyses of the *I(Q)* curves resulted in high quality linear plots with low residuals in two distinct regions (Fig. 5, *A* and *B*), not three regions as typically seen for IgG1 antibodies (8). The *Q.R*_*G*_ fit limits for the low *Q* region for the *R*_*G*_ values were satisfactory at 0.42 to 1.20 and 0.43 to 1.21 nm^-1^ (Fig. 5*A*). The Guinier fits gave a mean *R*_*G*_ value of 4.09 ± 0.08 nm for C5-Fc with glycans, and 4.06 ± 0.06 nm for C5-Fc without glycans (Table 2), these being the same within error of each other. The mean *R*_*XS*_ values were 1.69 ± 0.02 and 1.73 ± 0.02 (Table 2), these being taken from satisfactory *Q.R*_*XS*_ limits of 0.67 to 1.14 and 0.68 to 1.17 (Fig. 4). In contrast to the *R*_*G*_ values above, after deglycosylation, the *R*_*XS*_ values were increased by 0.04 nm at all concentrations. A similar increase in the *R*_*XS-1*_ value of 0.04 nm after deglycosylation was reported previously for human IgG1 (8).

**Table 2:** Experimental SAXS and SANS parameters for C5-Fc

Sample	Concentration (mg/ml)	R_G_ (nm)	R_XS_ (nm)	L (nm)	M1 (nm)	M2 (nm)
X-rays for C5-Fc with glycans	5.00	4.15 ± 0.14	1.69 ± 0.09	13.8	3.83	8.96
	4.00	4.13 ± 0.15	1.68 ± 0.12	13.4	3.87	8.81
	3.00	4.11 ± 0.14	1.66 ± 0.12	12.8	3.76	8.93
	2.00	4.11 ± 0.16	1.68 ± 0.15	12.8	3.75	9.02
	0.50	4.11 ± 0.37	1.69 ± 0.11	12.9	3.74	8.60
	1.00^a^	3.92 ± 0.14	1.71 ± 0.03	13.2	3.82	8.83
X-rays for C5-Fc without glycans	5.00	4.12 ± 0.11	1.72 ± 0.10	12.9	4.00	9.02
	4.00	4.12 ± 0.12	1.72 ± 0.11	13.3	4.00	9.04
	3.00	4.10 ± 0.17	1.73 ± 0.05	12.8	3.97	9.07
	2.00	4.00 ± 0.29	1.76 ± 0.08	13.0	3.97	9.46
	0.50	4.03 ± 0.42	1.73 ± 0.16	13.1	4.06	8.83
	1.00^a^	4.01 ± 0.14	1.72 ± 0.02	13.1	4.06	9.14
Neutrons for C5-Fc with glycans	6.00	4.29 ± 0.61	1.48 ± 0.12	13.0	4.18	8.47
	4.50	4.17 ± 0.47	1.51 ± 0.15	13.0	4.22	8.50
	3.30	4.15 ± 0.57	1.46 ± 0.20	12.3	4.21	8.54

a These two rows correspond to the size exclusion chromatography-SAXS runs.

Glycosylated C5-Fc was studied by SANS at 20^o^C in L-histidine buffer in D_2_O at concentrations of 3.3, 4.5, and 6.0 mg/ml. Again linear Guinier fits were seen (Fig. 5, *D* and *E*), giving a mean *R*_*G*_ value of 4.20 ± 0.08 nm. This value was similar to that from SAXS, but was a small increase of 0.11 nm compared to the X-ray values, although the errors were large at ∼0.5 nm (Table 2). The mean *R*_*XS*_ value was 1.48 ± 0.03 nm (Table 2), this being taken from satisfactory *Q.R*_*XS*_ limits of 0.45 to 0.89. This is typically seen for antibodies and corresponds to the reduced visibility (and contribution) of the hydration shell to the average cross-section shape of C5 and Fc regions.

The distance distribution function *P(r)* provides structural information on C5-Fc in real space after Fourier transformation of the experimental scattering curve *I(Q)*. The *P(r)* analyses produced *R*_*G*_ values that were similar to the Guinier analyses. By SAXS, the maximum length *L* was determined to be 13.1 ± 0.4 nm for C5-Fc with glycans and was unchanged at 13.0 ± 0.2 nm for C5-Fc without glycans (Fig. 5*C* and Table 2). In the *P(r)* curves, a single arrowed *M1* maximum corresponds to the most frequent interatomic distance r within C5-Fc. Unlike the *P(r)* curves for IgG antibodies, which generally display two peaks of similar sizes *M1* and *M2* (8), only one main peak *M1* was seen for C5-Fc. *M1* is assigned to the distances occurring with a single Fab or Fc region in IgG, thus its reappearance in C5-Fc is seen as expected. In IgG1, *M2* corresponds to the distances between the three Fab and Fc regions and monitors their separation. In C5-Fc, a shoulder peak *M2* was in fact observed that monitors changes in the mean separation of the C5 and Fc regions. In the X-ray scattering curve, the *M1* and *M2* peaks of glycosylated C5-Fc were 3.80 ± 0.05 nm and 8.9 ± 0.2 nm and were increased in deglycosylated C5-Fc to 4.01 ± 0.04 nm and 9.1 ± 0.2 nm. That change for *M1* may indicate an expansion of the Fc region following deglycosylation, while the *M2* values were unchanged within error. The corresponding neutron values of *L* of 12.8 nm, *M1* of 4.20 ± 0.02 nm, and *M2* of 8.5 ± 0.1 nm were similar to the X-ray values and confirmed these.

### Atomistic modeling of the C5-Fc solution structure

**Figure 6:**
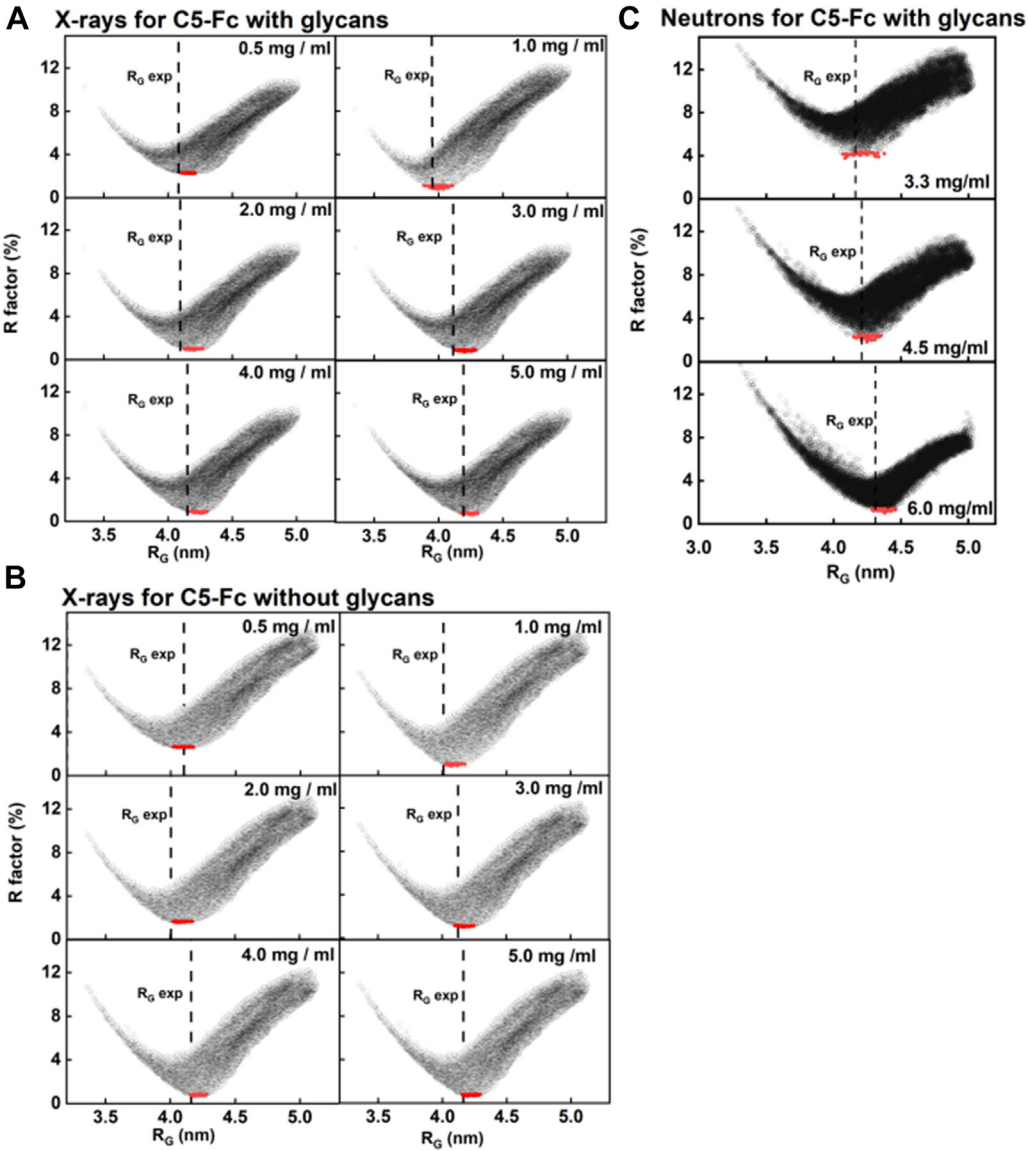
**Atomistic modeling analyses for the C5-Fc solution structure.** The *R*-factors for the 72,737 sterically acceptable models for the two-chain C5-Fc structure with glycans from the TAMC simulations are plotted against the *R*_*G*_ values for each model for X-rays (*A*) and neutrons (*C*). The *R*-factors for the 56,749 sterically acceptable models for C5-Fc without glycans from the TAMC simulations are plotted against the *R*_*G*_ values for each model for X-rays (*B*) only. The X-ray and neutron fits for different C5-Fc concentrations are shown. The experimental *R*_*G*_ is represented by a *dashed vertical line*. The minimum of each plot is close to the experimental *R*_*G*_ value in each panel. The top 100 best-fit C5-Fc models with the lowest *R*-factors are shown in *red circles*. TAMC, torsion angle Monte Carlo.

The atomistic modeling simulations of the scattering curves for the glycosylated and deglycosylated C5-Fc structures were performed to locate the two C5 regions relative to the Fc region (Fig. 1). The simulations were initiated from high-resolution crystal structures for C5 and the Fc region of human IgG1 and the C5-Fc sequence in Figure 2 (Experimental procedures). The C5 and Fc regions were joined by a 26-residue hinge peptide, built using Modeller (Experimental procedures). For the glycosylated C5-Fc models, two glycan chains were constructed and added to Asn^206^ in the C_H_2 domain. The three hinge disulfides were connected to follow the mass spectrometry showing their existence (Fig. 2*D*). Finally, both structures were energy minimized to create two full-length C5-Fc starting structures. The torsion angle Monte Carlo (TAMC) module in SASSIE-web was used to generate 72,737 glycosylated models and 56,749 deglycosylated C5-Fc models that were stereochemically realistic. These were loaded into the SasCalc module to generate the theoretical X-ray and neutron scattering curves. Comparisons with the X-ray and neutron experimental curves gave the R-factor *versus R*_*G*_ distributions for 15 concentrations (Fig. 6). There, all 15 analyses showed a V-shaped distribution with its minimum close to the experimentally observed *R*_*G*_ values. The 100 best-fit models were chosen based on having the lowest *R*-factors (red, Fig. 6). The range of these 100 *R*-factors for each concentration was low, this being 0.80 to 2.23% for the best-fit glycosylated models and 0.61 to 2.58% for the best-fit deglycosylated models (Table 3). This indicated high quality X-ray curve fits between the experimental and modeled curves.

**Table 3:** Modeling fits of the X-ray and neutron scattering data for C5-Fc

Sample	Concentration (mg/ml)	Range of *R*-factors (%)	*R*-factor for the best fit (%)	Averaged *R*-factors for the 100 best fits (%)	R_G_ for the best fit (nm)	R_G_ for the top 100 best fit (nm)	R_xs_ for the best fit (nm)	R_xs_ for the top 100 best fit (nm)
X-rays for C5-Fc with glycans	5.00	0.70–10.9	0.70	0.81	4.26	4.17–4.31	1.63	1.58–1.69
	4.00	0.76–10.7	0.76	0.86	4.26	4.15–4.31	1.63	1.58–1.70
	3.00	0.75–10.7	0.75	0.86	4.21	4.13–4.29	1.69	1.58–1.70
	2.00	0.89–10.8	0.89	0.99	4.20	4.12–4.27	1.68	1.59–1.71
	0.50	2.23–10.9	2.23	2.27	4.19	4.10–4.21	1.69	1.57–1.77
	1.00^a^	0.76–12.3	0.75	1.02	4.00	3.89–4.11	1.70	1.65–1.81
X-rays for C5-Fc without glycans	5.00	0.61–12.1	0.61	0.73	4.20	4.15–4.29	1.69	1.67–1.75
	4.00	0.66–12.3	0.66	0.76	4.18	4.15–4.28	1.73	1.66–1.75
	3.00	1.00–12.5	1.00	1.10	4.17	4.10–4.25	1.69	1.66–1.77
	2.00	1.56–13.1	1.56	1.63	4.06	4.02–4.17	1.75	1.69–1.81
	0.50	2.27–13.5	2.27	2.58	4.05	4.02–4.18	1.75	1.68–1.80
	1.00^a^	0.87–13.2	0.87	1.02	4.07	4.02–4.18	1.75	1.69–1.80
Neutrons for C5-Fc with glycans	6.00	1.36–15.8	1.36	1.61	4.34	4.23–4.38	1.67	1.55–1.69
	4.50	1.95–14.6	1.95	2.14	4.23	4.09–4.30	1.45	1.41–1.52
	3.30	3.11–14.6	3.11	3.53	4.25	4.14–4.34	1.42	1.40–1.53

a These two rows correspond to the size exclusion chromatography-SAXS runs.

**Figure 7:**
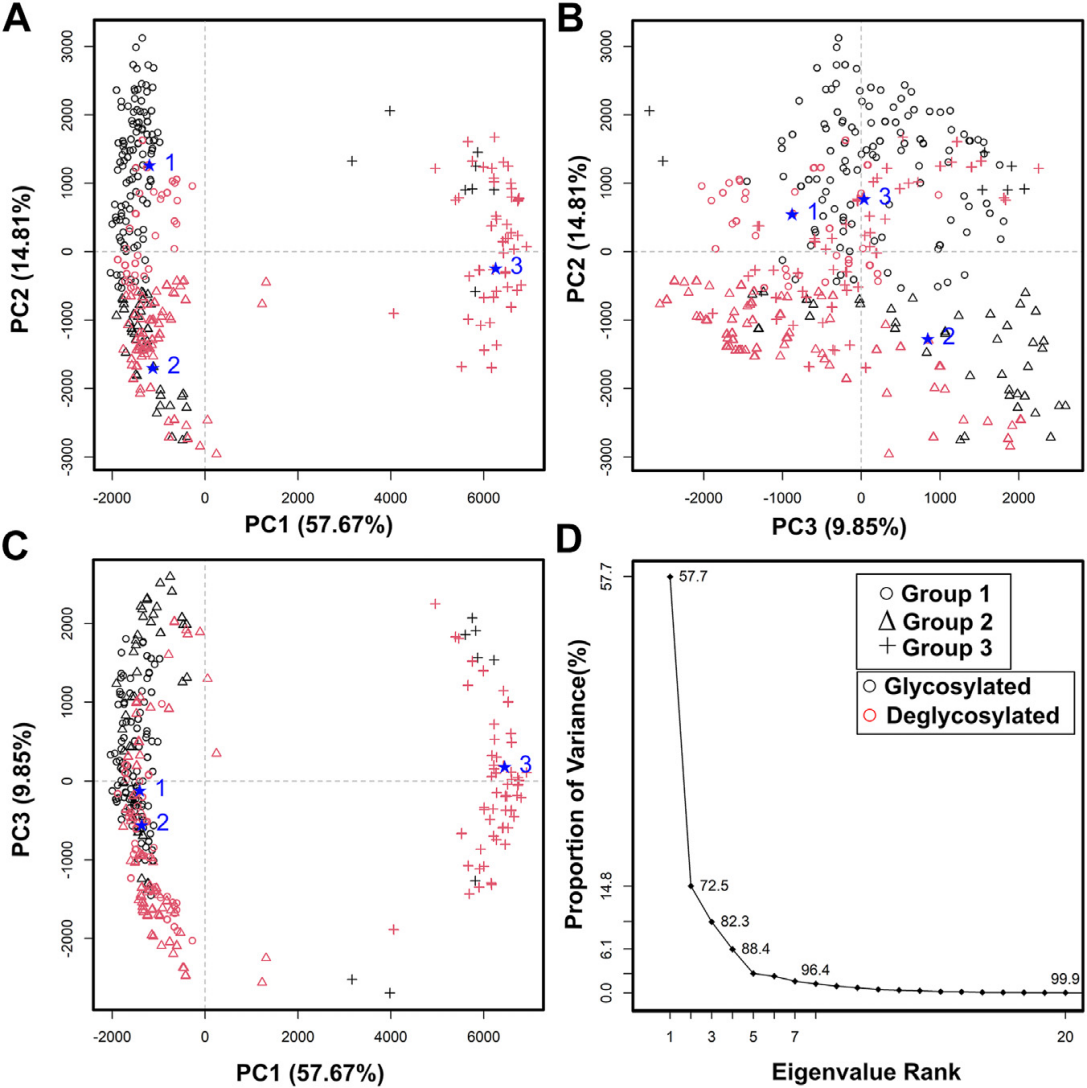
**Principal component analysis of the best-fit glycosylated and deglycosylated C5-Fc models.** Glycosylated models are represented in *black* and deglycosylated models are represented in *magenta*. In this, groups 1, 2, and 3 are represented by ○, Δ, and +, in that order, and the centroid model for each group is represented by large numbers (*blue*) and a ★. *A–D*, the six sets of 100 best-fit models from the experimental X-ray scattering curves for glycosylated and deglycosylated C5-Fc (Fig. 6, *A* and *B*) totaling 100 were grouped by PCA into five groups as shown in three panels *A–C* of PC2 *versus* PC1, PC3, *versus* PC2 and PC3 *versus* PC1. *D,* the first three eigenvalue rankings (PC1, PC2, and PC3) captured 82.3% of the variance in the 600 and 600 models. PCA, principal component analysis.

Principal component analysis (PCA) was performed on the 12 sets of 100 best-fit X-ray models that were identified from the lowest R-factors (Fig. 6) in order to identify any differences between the best-fit glycosylated and deglycosylated C5-Fc conformations (13). The PCA determines the correlated motions of protein residues in C5-Fc as linearly uncorrelated variables, each being termed a principal component (13). These “essential motions” were extracted from a covariance matrix of the atomic coordinates of the frames in the selected C5-Fc structure set. The eigenvectors of this matrix each have an associated eigenvalue that characterizes the clustering of the models based on structural coordinates (or variance). In order to eliminate bias in the PCA, the glycan chains were removed from the glycosylated C5-Fc models before comparison with the deglycosylated models. For the X-ray fits, the PCA indicated a difference between the glycosylated and deglycosylated C5-Fc models (black and magenta, respectively, Fig. 7 and Table 3). To determine the optimal number of clustered groups in the PCA, the elbow method involved plotting the total “within-cluster” sum of squares against the number of clusters. Thus, the sets of best-fit 100 glycosylated and 100 deglycosylated X-ray models were each optimally clustered into three distinct PCA groups (Table 4). The glycosylated models mostly occurred in the PCA groups 1 and 2, while the deglycosylated models mostly occurred in the PCA group 3 with some overlap with PCA group 2. The PCA thus differed between the two forms, indicating that these displayed different conformations.

**Table 4:** PCA analysis of the modeling fits for the X-ray data for C5-Fc

PCA Group	Filter	Model number	R_G_ (nm)	Centroid R_G_ (nm)	R_xs_ (nm)	Centroid R_xs_ (nm)
Group 1	Total	530	3.93–4.38	N.A.	1.66–1.81	N.A.
	Glycosylated	408	3.93–4.38	4.23	1.67–1.81	1.72
	Deglycosylated	122	4.02–4.30	4.13	1.66–1.81	1.73
Group 2	Total	462	4.38–3.93	N.A.	1.66–1.81	N.A.
	Glycosylated	178	3.93–4.38	4.19	1.66–1.80	1.73
	Deglycosylated	462	4.02–4.28	4.13	1.66–1.81	1.73
Group 3	Total	208	4.33–4.02	N.A.	1.66–1.81	N.A.
	Glycosylated	14	4.23–4.33	4.22	1.66–1.75	1.70
	Deglycosylated	194	4.02–4.28	4.17	1.66–1.81	1.72

N.A., not available.

**Figure 8:**
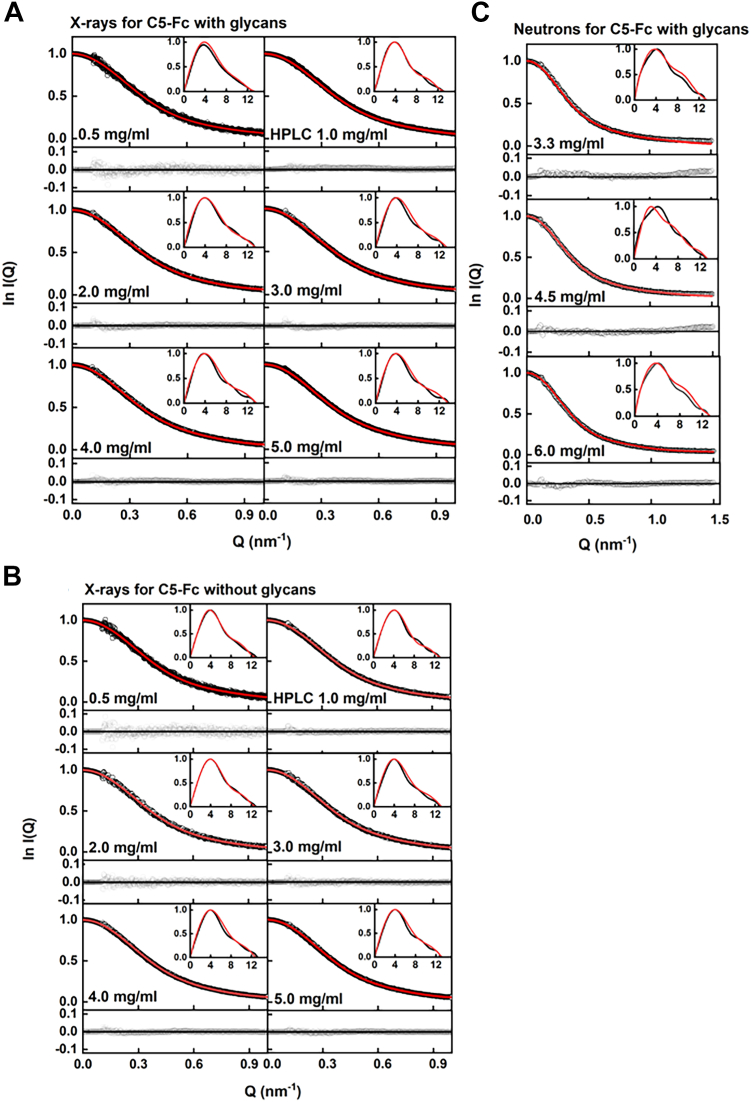
**Scattering curve fits to the experimental data for each of the best-fit C5-Fc models**. Each experimental *I(Q)* and *P(r)* curve is denoted by *black circles and lines,* respectively, and each best-fit modeled curve is denoted by *solid red lines*. The curve fit residuals are shown below each fit.

**Figure 9:**
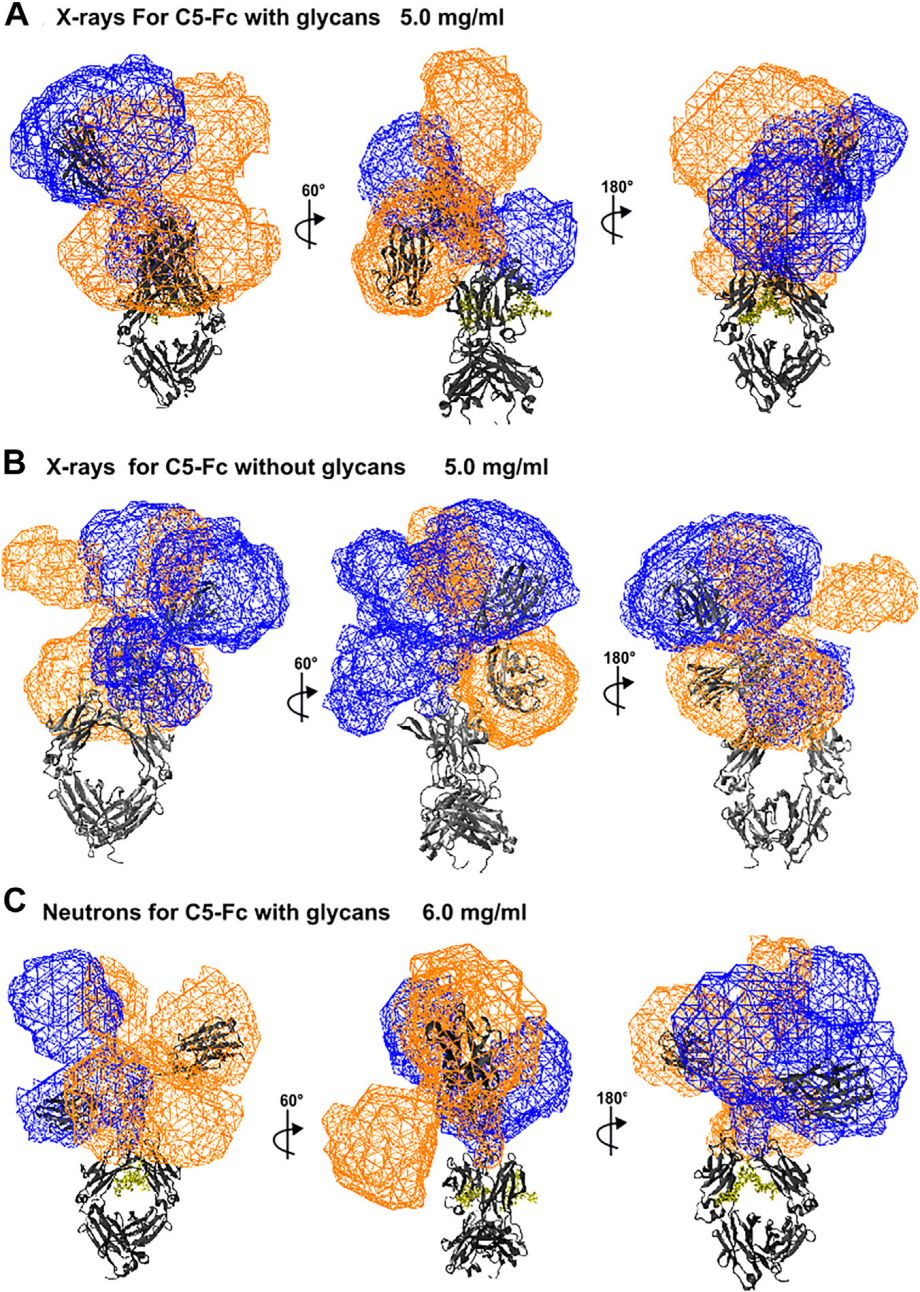
**Views of representative best-fit X-ray and neutron solution structures for C5-Fc.***A*–*C*, the 100 best-fit X-ray and neutron structures at each of three concentrations as specified are shown in three orientations that differ by rotations about their vertical axis. The *black ribbon cartoon* denotes the protein backbone of each best fit C5-Fc structure, with the Fc region shown at the base of each structure. The *blue* and *orange* wireframe envelopes denote the positions of the two C5 regions in the top 100 best-fit models. Bilobal envelopes are seen because no account was taken of the 2-fold symmetry of C5-Fc, meaning that both types of structures are visible. The glycans when present are shown in *yellow*.

The visually excellent ln *I(Q)* and *P(r)* X-ray and neutron curve fits, especially the agreement of the main peak in the experimental *P(r)* curves with that in the theoretical *P(r)* curves, confirmed the validity of the modelling fits (Fig. 8, *A–C*). The three sets of 100 best-fit X-ray and neutron models at their highest concentrations were used to generate wire-frame models, showing the location of the two C5 regions relative to the Fc region (Fig. 9). In all three cases, similar bilobal distributions were often observed for each of the two C5 regions in each C5-Fc structure, each of which were not in a fully extended orientation relative to the Fc region.

**Figure 10:**
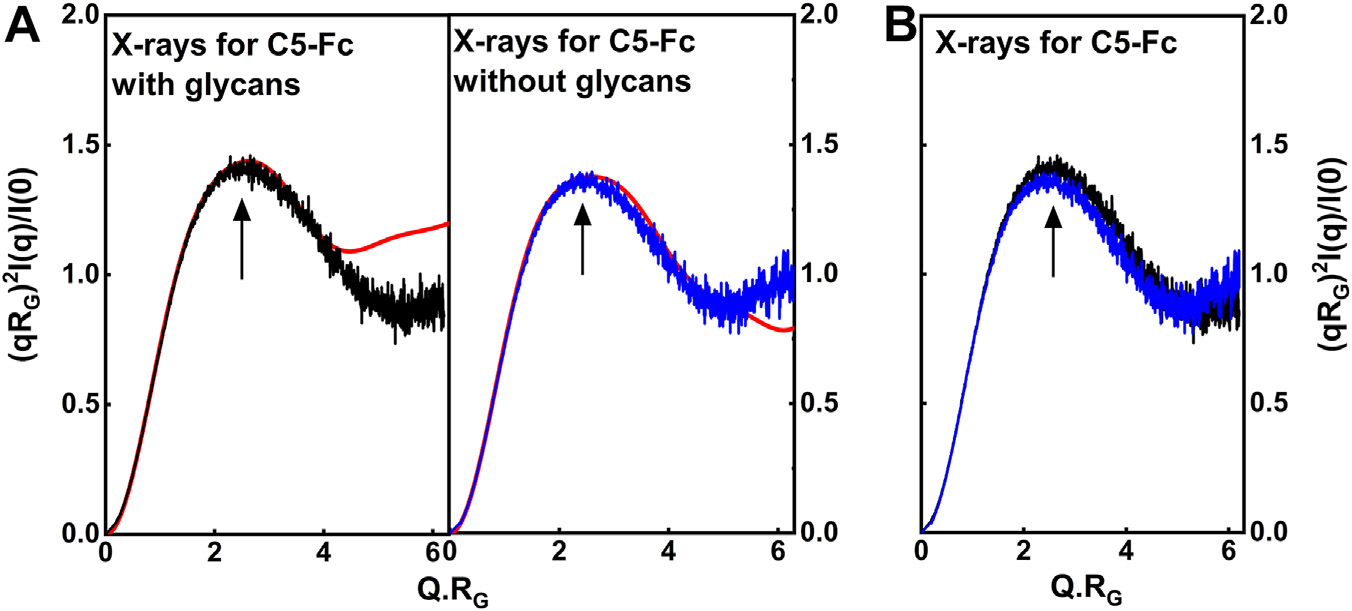
**Normalized Kratky plots for C5-Fc.***A*, in the X-ray scattering curves, the experimental curve concentrations are both 5 mg/ml. The X-ray curves corresponding to glycosylated and deglycosylated C5-Fc are shown in *black* and *blue*, respectively. The *red lines* represent the curves from the best-fit models. The main peak in each plot is *arrowed*. *B*, the two experimental curves corresponding to C5-Fc with and without glycan are overlaid.

The dimensionless Kratky analysis of (*Q.R*_*G*_)^2^.*I(Q*)/*I(0) versus Q.R*_*G*_ plots provides information on protein flexibility in solution. These plots show whether the macromolecule has a globular structure or possesses intrinsically disordered regions (14). Based on the X-ray experimental scattering curves for glycosylated and deglycosylated C5-Fc that showed excellent signal:noise ratios at large *Q*, the highest sample concentrations at 5.0 mg/ml and 5.0 mg/ml were used for the Kratky analysis in comparison with the corresponding best-fit modeled theoretical scattering curve. Unlike monoclonal IgG1, where two clear Kratky peaks were observed at *Q.R*_*G*_ values of around 2 and 4 (8), the Kratky plots for C5-Fc demonstrated only the first peak in both the experimental and theoretical modeled curves (Fig. 10*A*). The *Q.R*_*G*_ values for the experimentally glycosylated and deglycosylated peaks in the X-ray Kratky curves were 2.58 and 2.59. Given the similarity of the Kratky plots for both forms of C5-Fc (Fig. 10*B*), it was concluded that no pronounced flexibility or disorder in this protein was visible after deglycosylation.

As another test of the scattering modeling, the *s*^*0*^_*20,w*_ values for the six sets of best-fit 100 glycosylated C5-Fc models (Figs. 7 and 8) were calculated using HullRad (15). This gave an *s*^*0*^_*20,w*_ range of 4.22 to 4.50 S for the best-fit models from the six X-ray concentrations for glycosylated C5-Fc (Table 3). These values agreed well with the experimental *s*^*0*^_*20,w*_ values of 4.49 to 4.57 S for glycosylated C5-Fc in light water (Table 1). These agreements corroborated the outcome of the atomistic scattering modeling, given that the mean difference between the modeled and experimental values should typically be ± 0.21 S for related macromolecules (16).

## Discussion

Typically, full-length antibody structures are not readily crystallizable, thus little is often known about their solution structure. This AUC, SAXS, and SANS study, coupled with MC atomistic modeling, has determined solution structure of a recombinant full-length molecule C5-Fc protein that was verified using gel filtration, SDS-PAGE, and mass spectrometry. The AUC datasets showed that both C5-Fc and its deglycosylated form are monomers, each being formed from two heavy chains in L-histidine buffer in both light and heavy water (Fig. 1*A*) and showing no concentration dependences. These findings opened the way for detailed SAXS and SANS data collection on C5-Fc and its deglycosylated form to establish their Guinier *R*_*G*_ and *R*_*XS*_ values and their distance distribution curves *P(r)*. The ability to fit the SAXS and SANS data by atomistic scattering modeling using SASSIE (17) based on molecular dynamics and MC simulations gave excellent curve fits to stereochemically correct trial structures. The hinge with three S–S bridges for glycosylated and deglycosylated C5-Fc were identified by PCA to be fully formed, this being consistent with mass spectrometry. The clearest view of the final result was determined from wireframe representations of the 100 best-fit structures (Fig. 9). As one important outcome of this study, it was clear that the C5 regions in C5-Fc were revealed to be in sufficiently extended conformations to be able to bind the RBD of the SARS-CoV-2 coronavirus spike protein.

**Figure 11:**
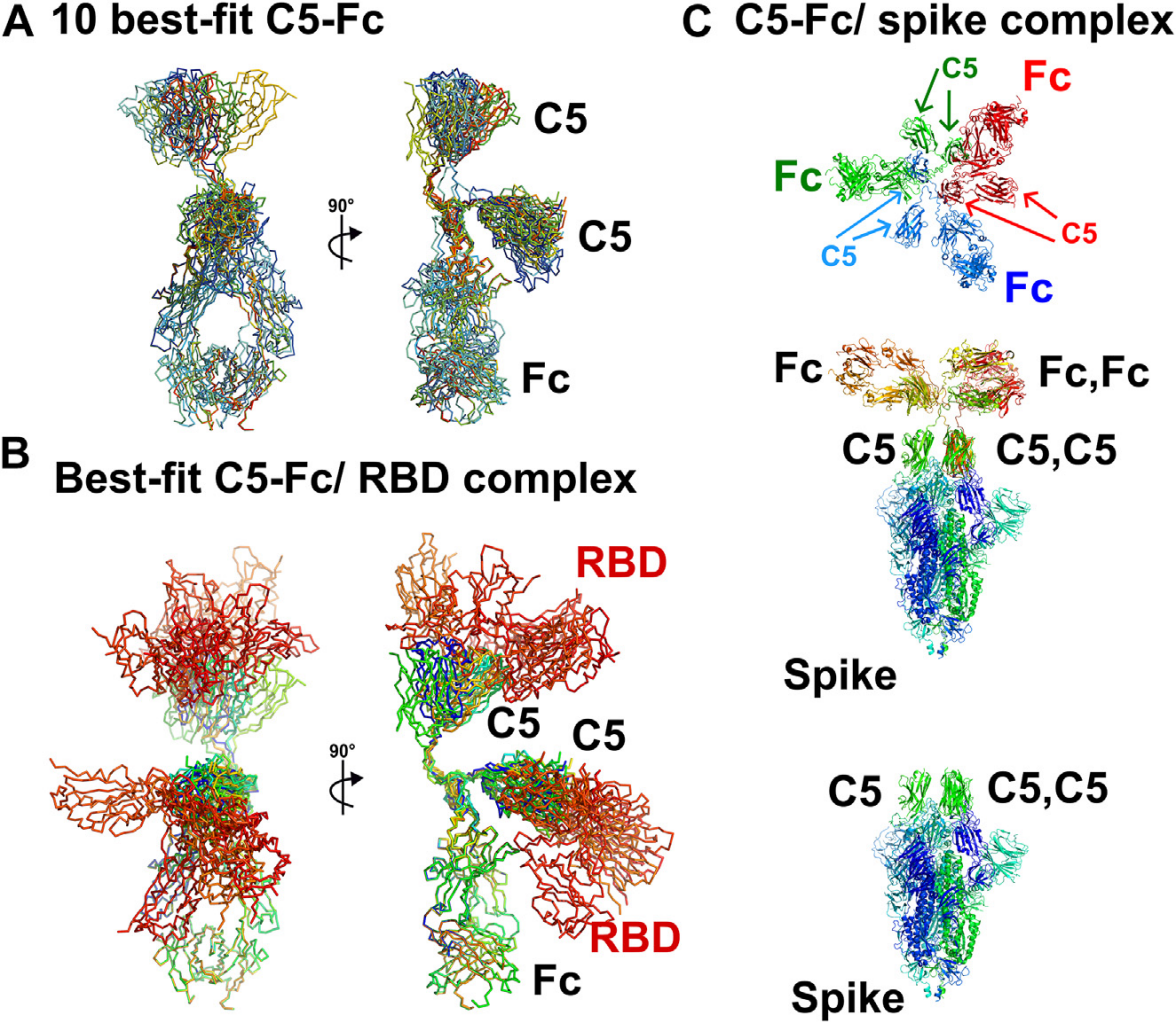
**Comparison of the ten best-fit glycosylated C5-Fc models and their complexes with the receptor-binding domain of the SARS-CoV-2 coronavirus spike.***A*, the eight C5-Fc best-fit and the two superimposed symmetry-related C5-Fc best-fit structures are shown, taken from the ten best-fit C5-Fc X-ray structures at 5 mg/ml. The view on the right is rotated by 90° about the vertical axis. *B*, the eight C5-Fc best-fit structures are shown in their complex with the RBD (*red*) from the SARS-CoV-2 coronavirus spike protein. The superimpositions were performed using the crystal structure of the C5-RBD complex (PDB ID: 7OAO). *C*, views of the putative complex of C5-Fc from (A) with the C5–spike complex (PDB ID: 7OAN), At the *bottom*, a side view of the 7OAN structure is shown with the three C5 nanobodies (*green*) bound to the RBD at the *top* of the spike. In the center, three C5-Fc molecules are shown with the top-most C5 region from (*A*) superimposed onto the three C5 molecules at the *top* of the spike. At the *top*, only the three C5-Fc molecules are shown in a *top view* to indicate that there is no steric overlap between the three C5-Fc molecules in this modeled complex. RBD, receptor-binding domain.

Two sets of conformational best-fit structural locations for C5 in the full structure were detected in the modeling fits. Rotations of 180° about the long axis of the Fc region showed that the two sets of structures were equivalent to each other, which is as expected given the 2-fold symmetry of the two heavy chains in the C5-Fc structures (Fig. 11*A*). These were effectively two copies of the same structure related by a symmetry axis. The top 10 best-fit structures for unbound and bound C5-Fc, and correcting for the 180° equivalence of structures shows that both the C5 domains in C5-Fc were exposed to solvent. This solvent exposure means that both C5 domains are fully available for binding to the RBD of the spike protein without conformational rearrangement. A subaim of this work was to evaluate further the effect of removing the conserved glycans in the Fc region. We had previously examined this for human IgG1, IgG3, and IgG4 to show that comparatively large changes could be seen for human IgG1, but less so for IgG3 and IgG4 (8, 18, 19). The advantage of the present work is that the Fc region comprises two-thirds of the C5-Fc structure and changes in this are more easily seen. The present C5-Fc study however showed that little change has been seen, following deglycosylation, akin to the behavior seen with IgG3 and IgG4.

Importantly, the solution structure of C5-Fc provided new insight into how the molecule may interact with the spike protein and hence neutralize the virus in infection assays. The RBD is the main immuno-dominant region of the spike protein and the target for neutralizing antibodies generated either by vaccination or infection. The C5 moiety recognizes a site that overlaps with the ACE2 binding site on the top of the RBD (4). Previously, structures of the C5 monomer bound to both the isolated RBD and a stabilized prefusion trimer had been determined by X-ray crystallography and cryo-EM, respectively (4). The cryo-EM structure (PDB ID: 7OAO) showed that a C5 nanobody is bound to each RBD of the trimeric spike protein in an all-down conformation, leading to the speculation that C5 binding stabilizes a closed state preventing the availability of at least one RBD for ACE-2 binding. The complementarity-determining regions of the C5 VHHs are approximately 5 to 6 nm apart in the C5-Fc solution structure (Fig. 11*A*). Comparison with this C5:spike complex indicates that for steric reasons one bivalent C5-Fc molecule is not able to bind simultaneously to two RBDs of the same spike protein, the latter being estimated from the cryo-EM structure (PDB ID: 7OAN) to be spaced approximately 4 nm apart (4). It is however possible for three C5-Fc molecules to bind simultaneously to the same spike protein through the uppermost C5 molecule of C5-Fc as illustrated in Figure 11*A*. As shown in Figure 11*C*, three molecules of C5-Fc are able to neutralize all three RBDs of the spike protein through its monovalent binding as shown. This would facilitate its high affinity. The majority of nanobodies developed against the spike protein of SARS-CoV-2 coronavirus bind to the RBD; to gain binding avidity, these have been expressed as dimeric Fc fusion proteins for viral neutralization tests. Therefore, our study of C5-Fc offers a general model for how these nanobody-derived fusion proteins are able to interact with the virus spike, leading to neutralization of the virus.

The advantage of our combined SAXS-SANS-AUC-MC approach is the opportunity to determine full-length antibody structures, this being the functional structure in distinction to the commonly studied Fab or Fc fragments. Traditionally solution scattering analyses are at low resolutions of around 2 nm, while protein crystallography routinely achieves 10-fold better resolutions. The atomistic modeling of the SAXS and SANS data gives improved analyses compared to previous because of the ability to use known crystal structures to constrain the fits of the scattering curves. Our recent studies have resulted in molecular solution structures for all four human IgG subclasses, IgG1-IgG4 (8, 18, 19, 20). The present study extends the methodology to llama-sourced heavy-chain antibodies, and again full atomistic structures have been determined. Interestingly, the methodology is able to detect the effect of deglycosylation on the antibody structure. Changes in the Fc region to become more disorganized in deglycosylated human IgG1 were detectable by the SAXS-SANS-AUC-MC approach (8). Previous NMR solution studies of the glycosylated and deglycosylated Fc region (21) showed that its two C_H_2 domains were oriented by the glycans for optimal binding affinity with receptors. Crystallography of the deglycosylated Fc region showed that the C_H_2 domains had reorientated themselves into a more compact structure (22). These effects were less clear in human IgG3 and IgG4 (18, 19), and likewise in the present study for C5-Fc. These previous studies on the Fc region alone complement our results showing that the Fc region within intact C5-Fc is more flexible after deglycosylation. Overall, the atomistic modeling approach in combination with high quality SAXS data with little noise at large *Q* values has been of great value in studying structural perturbations in antibodies caused by the removal of its two glycans.

## Experimental procedures

### Purification and composition of C5-Fc

Recombinant C5-Fc was produced by transient expression in mammalian Expi293 cells and purified by a combination of a HiTrap MabSelect SuRe Protein A column (Cytiva) and Superose 6 gel filtration in PBS (137 mM NaCl, 8.1 mM Na_2_HPO_4_, 2.7 mM KCl, and 1.5 mM KH_2_PO_4_, pH 7.4) (4). Yields were around 65 mg/l of culture medium. C5-Fc was deglycosylated by enzymatic cleavage using PNGase F (New England Biolabs) to remove the N-linked oligosaccharides from the Fc region (23, 24). One milliliter of 3 mg/ml native C5-Fc was mixed with 3 μl of 10,000-unit PNGase F and incubated for 10 h at 37 °C, by which deglycosylation proceed to completion (8). The deglycosylated C5-Fc was then recovered by gel filtration to remove the unwanted PNGase F. Prior to measurements (below), gel filtration, SDS-PAGE, and mass spectrometry were used to verify the purity of C5-Fc and its deglycosylation. The C5-Fc samples were passed through a Superose 6 Increase 10/300 GL column (Cytiva) on an AKTA GO (Cytiva) to remove trace nonspecific aggregates. For SDS-PAGE, TCEP; Thermo Fisher Scientific) was used as a reducing agent with NuPAGE 4 to 12% Bis-Tris Gel (Invitrogen). The C5-Fc sequence (Fig. 2) gave the molecular weight of C5-Fc without glycan as 78,318 Da with a partial specific volume $\bar{v}$ of 0.732 ml/g. The molecular weight of C5-Fc with two glycans was calculated as 81,206 Da with a partial specific volume $\bar{v}$ 0.729 ml/g (11). The C5-Fc protein concentration was determined by the absorbance reading at 280 nm and using absorption coefficients of 12.9 and 13.4 (1%, 280 nm, 1 cm path length) for glycosylated and deglycosylated C5-Fc, respectively (11).

For mass spectrometry, C5-Fc in 10 mM L-histidine, 138 mM NaCl, and 2.6 mM KCl was concentrated, and buffer exchanged into PBS with Amicon Ultra-0.5 30 kDa cut-off centrifugal filters (Sigma-Aldrich) to 0.6 mg/ml. This was reduced with 20 mM TCEP and 2-mercaptoethylamine-HCl (2-MEA). The 2-MEA-reduced protein was next labelled with a 100 × molar excess of EZ-Link maleimide-PEG-2 biotin stock (Thermo Fisher Scientific) in PBS (25). All native, maleimide-labeled, TCEP-, and 2-MEA–reduced proteins were then buffer exchanged at 0.2 mg/ml 2 h in advance into 100 mM ammonium acetate with Zeba desalting columns (Thermo Fisher Scientific) for mass spectrometry on a 6510 quadrupole time-of-flight LC/MS system (Agilent Technologies). The sample (8 μM) was then injected onto a PLRP-S, 1000 A, using a 150 mm × 2.1 mm column at a volume of 10 μl. The column was maintained at 60 °C at a flow rate of 0.3 ml/min. The separation was achieved using mobile phases A (water with 0.1% formic acid) and B (acetonitrile, with 0.1% formic acid) using a gradient elution. The column effluent was continuously electrosprayed into the capillary electrospray ionization source of the Agilent 6510 mass spectrometer, and electrospray ionization mass spectra were acquired in positive electrospray ionization mode using the *m/z* range 1000 to 3200 in profile mode. The raw data were analyzed in Mass Hunter software version B.07.00 (https://www.agilent.com/cs/library/usermanuals/public/G3335-90215_MassHunter_Offline_Installation-en.pdf) with a built-in protein deconvolute function to convert zero-charge mass spectra to intensity/mass plots.

### AUC sedimentation velocity data and analysis for C5-Fc

Native C5-Fc after gel filtration was concentrated to 6 mg/ml using Amicon Ultra 3 ml spin concentrators (50-kDa cut-off). This was dialyzed at 4 °C into 20 mM L-histidine, 138 mM NaCl, 2.6 mM KCl, pH 6, in H_2_O and D_2_O overnight with three buffer exchanges. This histidine buffer was found to increase the solution stability of antibodies and collagens and reduce any propensity to form aggregates (8, 26). Dialyzed samples were diluted in a concentration series from 6 mg/ml to 1 mg/ml. The solvent density and viscosity were measured on an Anton Paar DMA 5000 density meter and an Anton Paar Lovis microviscometer (Anton Paar) at 20 °C. The solvent density in H_2_O was 1.00380 g/ml, and the viscosity was 1.040 mPa s. In D_2_O, the solvent density and viscosity were 1.11106 g/ml and 1.2745 mPa s (11). The samples and their reference buffer were loaded into the AUC cells. The difference in absorbance and interference between the reference buffer and the sample was measured on an Optima AUC instrument (Beckman Coulter) with AnTi 50 rotors at 20 °C at a rotor speed of 40,000 rpm. The absorbance and Rayleigh interference datasets for C5-Fc were acquired simultaneously during the 12-h run. The resulting 650 scans for each concentration were loaded into SEDFIT (version 16.36; https://sedfitsedphat.github.io/) for fits using the continuous *c(s)* distribution model with a resolution of 250 in a sedimentation coefficient s_20,w_ range of 0 to 15 S (27, 28). For the fits, the overall RMSD was judged to be adequate if less than 0.02, following the adjustment of parameters such as the positions of the meniscus and cell bottom, the baseline and the frictional ratio *f/fo*. The resulting size-distribution *c(s)* plot gives the sedimentation coefficient(s) s_20,w_ for the species present.

### SAXS and SANS data and analyses for C5-Fc

SAXS data were obtained on Instrument B21 in one session (September 2022) at the Diamond Light Source at the Rutherford Appleton Laboratory (Didcot) in batch-SAXS and size exclusion chromatography-SAXS (SEC-SAXS) modes (29). The beamline B21 operated at an energy of 12.4 keV with a 2-m sample-to-detector (Dectris) distance of 4.01 m. The detector has a resolution of 1475 × 1679 pixels (pixel size of 172 × 172 mm) and a *Q* range of 0.04 to 4 nm^-1^(where *Q* = 4 π sin *θ*/λ; 2*θ* = scattering angle; λ = wavelength). A scattering reference buffer was used to calibrate the intensity to absolute units. Predialyzed samples of native C5-Fc and deglycosylated C5-Fc (0.5–5.0 mg/ml) in 10 mM L-histidine, 138 mM NaCl, 2.6 mM KCl, in light water were loaded onto a 96-well plate. In the batch-SAXS runs, an automatic sampler injected 35 μl of sample from the well plate into a temperature-controlled quartz cell capillary with a diameter of 1.5 mm. Datasets of 30 frames with a frame exposure time of 1 s each were acquired in duplicate as a control of reproducibility. Frames were subtracted from the reference buffer and averaged after programmed inspection for minimum radiation damage (30, 31). For the SEC-SAXS runs, 50 μl of sample from the well plate were loaded onto a KW402.5 (Shodex) HPLC column connected to an Agilent HPLC system (Agilent). For SEC-SAXS, ScÅtter (version VI) was used for HPLC buffer subtraction, data reduction, and normalization by concentration (29).

SANS data on native C5-Fc in heavy water were obtained on instrument SANS2D at the ISIS pulsed neutron source in one session (July 2022) at the Rutherford Appleton Laboratory (32). Native C5-Fc (3.3 mg/ml, 4.5 mg/ml, and 6 mg/ml) predialyzed samples in 10 mM L-histidine, 138 mM NaCl, and 2.6 mM KCl in heavy water were loaded into 1 ml, 2-mm path length banjo-shaped cells in a thermostatted sample rack at 20 °C. SANS2D neutron data were recorded with 4 m of collimation, a 4 m sample-to-detector distance, a 12 mm sample aperture, and a wavelength range of 0.175 to 1.65 nm made available by the time-of-flight method. This gave a *Q* range of 0.05 to 4 nm^−1^. The data were acquired using a two-dimensional ^3^He detector with 512 × 512 pixels of 7.5 × 7.5 mm^2^ in size. The raw data were reduced, merged, and subtracted using MANTID software (https://www.mantidproject.org/).

The SAXS and SANS scattering curves *I(Q)* were analyzed in the SCT suite of programs (33) and ATSAS (version 3.1.3; https://www.embl-hamburg.de/biosaxs/software.html) (34). The data provided the *R*_*G*_, cross-sectional radius of gyration (*R*_*XS*_), and maximum dimensions. Guinier analyses of the *I(Q)* curve at low *Q* ranges yield the *R*_*G*_ value, which is the average solution structural elongation from the center of the protein, and the forward scattering at zero angle I(0) (35). For C5-Fc, the *Q.R*_*G*_ range was taken up to 1.3 in order to determine satisfactory Guinier values:

$$\ln\;I\left(Q\right)=\ln\;I\left(0\right)-\frac{{R_{G}}^{2}Q^{2}}{3}$$

For an elongated protein such as C5-Fc, the mean *R*_*G*_ of the cross-sectional structure R_XS_ was determined from the equation below in a larger *Q* fit range:

$$\ln\left[I\left(Q\right)Q\right]={\left[I\left(Q\right)Q\right]}_{Q\to 0}-\frac{{R_{XS}}^{2}Q^{2}}{2}$$

The indirect Fourier transformation of the scattering curve *I(Q)* in reciprocal space into real space to give the distance distribution function *P(r)* was carried out using the program GNOM (36, 37):

$$P\left(r\right)=\frac{1}{2\pi^{2}}\int_{0}^{\infty }I\left(Q\right)Qr\;\sin\left(Qr\right)dQ$$

The *P(r)* curve denotes the distribution of all interatomic distances r between all volume elements in the macromolecule. The *P(r)* curve gives *L* as the largest dimension for a macromolecule and *M1* and *M2* as the most common distance vectors within C5-Fc.

### Atomistic modeling of C5-Fc

C5-Fc is comprised of two llama-derived single-domain nanobodies C5 and a glycosylated Fc region (Fig. 1). To create the starting C5-Fc structure, the molecular structure of C5 was extracted from the crystal structure of the C5–RBD complex (PDB ID: 7OAO), based on the sequence alignment of Figure 2. The native and deglycosylated Fc region and hinge were obtained from the best-fit human IgG1 solution structure from our earlier study (8). That Fc structure had originated from the rituximab IgG1 antibody Fc crystal structure (PDB ID: 4W4N) (38). The C5-Fc hinge region with the sequence ^**125**^EPKSCDKTHTCPPCPAPELLGGP^**147**^ between the C5 and Fc regions was structurally completed using MODELLER (version 10.3; https://salilab.org/modeller/) (39). In order to add the Fc glycans, the size of the two N-linked oligosaccharides at Asn^206^ on the C_H_2 domains were determined from the molecular mass difference before and after PNGase F cleavage. Two complex-type biantennary oligosaccharide structures with an Man_3_GlcNAc_2_Fuc_1_ core and two NeuNAc antenna was built and attached to the Fc region at Asn^206^ using the CHARMM-GUI server Glycan Reader tool (40, 41) (Fig. 1*A*). The complete Fc-glycan structure was then energy minimized using NAMD software (https://www.ks.uiuc.edu/Research/namd/) for 1 ns to achieve a relaxed structure, while ensuring no steric clashes with the Fc residues (19, 20). The three disulfide bridges in the hinge at Cys ^129^, Cys^135^, and Cys^138^ were linked using the CHARMM-GUI PDB Reader tool. The CHARMM force field parameters and protein structure file, including those for the disulfide bridges and glycans, were generated using the CHARMM-Gui Glycan Reader tool which is compatible with the CHARMM36 force field (39, 40, 41, 42, 43, 44). For each C5-Fc model, Glycan Reader was used to generate the CHARMM force field and PSF file, which was energy minimized for 50,000 steps using the simulation engine NAMD version 2.14 with the CHARMM36 force field (40, 44).

The two starting C5-Fc structures (with and without glycans) were now ready for the MC simulations of a library of physically realistic C5-Fc structures. To match the format requirement for the TAMC module in SASSIE-web, bash scripts were used to convert the nomenclature and numbering of the glycan and protein atoms in C5-Fc. For TAMC, six flexible regions were assigned within the hinges for both heavy chains, namely ^125^EPK^127^, ^131^ KTH^133^, and ^140^APE^142^ (Fig. 1, *B* and *C*). The rest of the C5-Fc structure was held rigid. For each of these nine linker residues per heavy chain, the backbone phi (φ) and psi (ψ) torsion angles were varied in steps of up to 180°. For the flexible region ^125^EPK^127^ which is close to the Fc region, the two Fc chains become mobile and can be structurally varied to create the required trial C5-Fc conformers for testing against the experimental scattering curve. For the other two flexible regions ^131^ KTH^133^ and ^140^APE^142^, these were programmed to retain the newly formed three disulfide bonds inside the hinge core, but would still create conformational variation during the MC simulations. The Fc region that is C terminal after the hinge was left unchanged because it was already the best-fit structure from our earlier MC study (8).

In the TAMC simulations to generate trial structures (45), a large number of attempted moves were not accepted because they were physically unrealistic. Only the sterically accepted moves were saved for further fit analyses. For the C5-Fc simulations with glycans, 450,000 moves were attempted, from which 72,737 models were accepted (16%). For the deglycosylated C5-Fc simulations, 400,000 moves were attempted, from which 56,794 models were accepted (14%). A theoretical scattering curve was generated from each accepted model using the SasCalc module in SASSIE-web (46, 47, 48). SasCalc calculated the scattering curve using an exact all-atom expression for the scattering intensity in which the orientations of the *Q* vectors are taken from a quasi-uniform spherical grid generated by the golden ratio (46). The golden ratio was set to be 33 for X-rays and 35 for neutrons after SasCalc test runs. For comparison with experiment, the extrapolated experimental curves from X-rays and neutrons were interpolated to zero *Q* using MATLAB (The MathWorks) in order to meet the input requirement for the *R*-factor comparison module in SASSIE-web. After interpolation, the *Q* spacing was uniform between the data points, and extrapolation extended the full experimental *I(Q)* curve to zero *Q*. Individually, this procedure produced 771 *Q* values between 0.0 and 1 nm^-1^ for the SAXS curve and 152 *Q* values between 0.0 and 1.5 nm^-1^ for the SANS curve.

For each theoretical scattering curve generated for accepted models of C5-Fc with and without glycans, the curve was compared with the X-ray and neutron interpolated experimental scattering curves, using the *R*-factor function in SASSIE-web for statistical analyses:

$$R=\frac{\sum \left\|\|I_{Expt}\left(Qi\right)\|-\eta \;\|I_{Model}\left(Qi\right)\|\right\|}{\sum \left\|I_{Expt}\left(Qi\right)\right\|}\times 100$$

where Qi is the *Q* value of the *i*^th^ data point; $I_{Expt}\left(Qi\right)$ is the experimental scattering intensity; and $I_{Model}\left(Qi\right)$ is the theoretical modelled scattering intensity (33). Lower *R*-factor values represent better fits. The 72,737 glycosylated and 56,749 deglycosylated C5-Fc models gave an *R*-factor *versus* R_G_ distribution based on comparisons with experimental data at different concentrations. For each concentration, the 100 best-fit models with the smallest *R*-factors were selected, and the best-fit model being that with the smallest *R*-factor. The wireframe models of the best-fit models were generated using SASSIE-web (https://sassie-web.chem.utk.edu/docs/overview/sassie_introduction.html) and PyMol (https://pymol.org/2/).

For the AUC modeling, the theoretical *s*_*20,w*_ values for the best-fit C5-Fc models were calculated directly from the atomic coordinates using HullRad (16). HullRad includes glycan residues for glycosylation, however there are inconsistencies in the Protein Database nomenclature for glycans. The nomenclature in the HullRad script was thus modified to ensure that the C5-Fc glycosylation was correctly incorporated in the *s*_*20,w*_ calculation.

## Data availability

All data are contained within this manuscript. The 100, 10, and single best-fit models for C5-Fc with glycans and C5-Fc without glycans that correspond to the X-ray fit searches at 5 and 5 mg/ml and the neutron best-fit searches at 6 mg/ml (Figs. 9 and 10) are available in supporting information. The single best-fit C5-Fc models were also deposited in the SASBDB database (https://www.sasbdb.org/) with the reference codes SASDSD2 (glycosylated) and SASDSE2 (deglycosylated). (links:

https://www.sasbdb.org/data/SASDSD2

https://www.sasbdb.org/data/SASDSE2)

## Supporting information

This article contains supporting information.

## Conflict of interest

The authors declare that they have no conflicts of interest with the contents of this article.

## Supporting information

SANS_C5_Fc_Glycosylated_6.0mgml_interpolated_normal_expt.dat

SANS_C5_Fc_Glycosylated_6.0mgml_Top1_best_fit

SANS_C5_Fc_Glycosylated_6.0mgml_Top1_best_fit_theoretical.dat

SANS_C5_Fc_Glycosylated_6.0mgml_Top10_best_fit

SANS_C5_Fc_Glycosylated_6.0mgml_Top100_best_fit

SAXS_C5_Fc_Deglycosylated_5.0mgml_interpolated-normal_expt.dat

SAXS_C5_Fc_DeGlycosylated_5.0mgml_Top1_best_fit

SAXS_C5_Fc_DeGlycosylated_5.0mgml_Top1_Best_fit_theoretical.dat

SAXS_C5_Fc_DeGlycosylated_5.0mgml_Top10_best_fit

SAXS_C5_Fc_DeGlycosylated_5.0mgml_Top100_best_fit

SAXS_C5_Fc_Glycosylated_5.0mgml_interpolated-normal_expt.dat

SAXS_C5_Fc_Glycosylated_5.0mgml_Top1_best_fit

SAXS_C5_Fc_Glycosylated_5.0mgml_Top1_Best_fit_theoretical.dat

SAXS_C5_Fc_Glycosylated_5.0mgml_Top10_best_fit

SAXS_C5_Fc_Glycosylated_5.0mgml_Top100_best_fit
